# Applying Artificial Intelligence Methods for the Estimation of Disease Incidence: The Utility of Language Models

**DOI:** 10.3389/fdgth.2020.569261

**Published:** 2020-12-15

**Authors:** Yuanzhao Zhang, Robert Walecki, Joanne R. Winter, Felix J. S. Bragman, Sara Lourenco, Christopher Hart, Adam Baker, Yura Perov, Saurabh Johri

**Affiliations:** Babylon Health, London, United Kingdom

**Keywords:** natural language processing, disease incidence, health statistic data, deep learning, machine learning

## Abstract

**Background:** AI-driven digital health tools often rely on estimates of disease incidence or prevalence, but obtaining these estimates is costly and time-consuming. We explored the use of machine learning models that leverage contextual information about diseases from unstructured text, to estimate disease incidence.

**Methods:** We used a class of machine learning models, called language models, to extract contextual information relating to disease incidence. We evaluated three different language models: BioBERT, Global Vectors for Word Representation (GloVe), and the Universal Sentence Encoder (USE), as well as an approach which uses all jointly. The output of these models is a mathematical representation of the underlying data, known as “embeddings.” We used these to train neural network models to predict disease incidence. The neural networks were trained and validated using data from the Global Burden of Disease study, and tested using independent data sourced from the epidemiological literature.

**Findings:** A variety of language models can be used to encode contextual information of diseases. We found that, on average, BioBERT embeddings were the best for disease names across multiple tasks. In particular, BioBERT was the best performing model when predicting specific disease-country pairs, whilst a fusion model combining BioBERT, GloVe, and USE performed best on average when predicting disease incidence in unseen countries. We also found that GloVe embeddings performed better than BioBERT embeddings when applied to country names. However, we also noticed that the models were limited in view of predicting previously unseen diseases. Further limitations were also observed with substantial variations across age groups and notably lower performance for diseases that are highly dependent on location and climate.

**Interpretation:** We demonstrate that context-aware machine learning models can be used for estimating disease incidence. This method is quicker to implement than traditional epidemiological approaches. We therefore suggest it complements existing modeling efforts, where data is required more rapidly or at larger scale. This may particularly benefit AI-driven digital health products where the data will undergo further processing and a validated approximation of the disease incidence is adequate.

## 1. Introduction

Accurate, comprehensive estimations of global health statistics are crucially important for informing health priorities and health policy decisions at global, national, and local scales (1). However, obtaining accurate and informative estimates of disease incidence and prevalence requires a substantial amount of time, money and expertise to design rigorous data collection processes, gather data, and build infrastructure for data collection. This is particularly challenging in developing countries where health systems have less capacity. More recently, comprehensive data on the incidence of different diseases in different settings have become an important component of AI-driven digital health products addressing global healthcare needs, which is difficult to achieve if data availability is limited.

Whilst collecting high quality data remains an important public health priority, sometimes rapid decision making is needed. Emerging diseases and medical advances for instance new drugs or vaccines, are two examples whereby public health priorities shift rapidly and policy makers cannot wait for the data for thoroughly evidence-based decisions. Policy decisions are then made using the best available knowledge, such as data from similar settings, data with known biases, or local expert opinion. Machine learning models have also used this information to impute estimates of disease incidence or prevalence much more quickly.

A “class” of machine learning techniques called deep neural networks (more broadly, deep learning algorithms) have recently seen a tremendous rise in their adoption across various fields (2). However, they require large “training” datasets to achieve high predictive performance, limiting their predictive ability when such data is not readily available. Large amounts of information about diseases exist online in free text and other unstructured formats, which has led to an increasing interest in the use of methods from Natural Language Processing (NLP), called language models, for healthcare.

Language models are widely used, for example in predictive text (3) and language translation (4). They estimate a probability distribution over a set of words (semantics and syntax), to compute the likelihood of some text occurring, given an input sequence. In order for language models to process and understand natural language, free-text words (or whole sentences) are converted into numeric values; referred to as word embeddings (or dense representations), which encode contextual information and meaning. The quality of these embeddings will be dependent on the underlying mechanics of the transformation and on the original text, which affects the utility of the embeddings for downstream tasks. This makes word embeddings especially useful for healthcare, since pre-trained embedding models obtained from publicly available biomedical text and data can be exploited for a variety of tasks.

The effectiveness of language models has been demonstrated on general language problems, such as question answering (5) and sentiment analysis (6). In medical contexts, deep learning approaches have been used for for diagnosis (7), disease clustering (8) and temporal modeling of electronic health records (9–13). Word embeddings have been explored for automated disease cohort selection (14), predicting hospital readmission from clinical notes (15) and for automated radiology report annotation (16). However, their use has never been explored in an epidemiological application for estimating disease incidence.

In this study, we evaluated the utility of using different pre-learned language models [GloVe (17), BioBERT (18), and the Universal Sentence Encoder [USE] (19)] to train disease incidence predictive networks. These language models compute a vectorized representation of free text inputs. When transforming disease and country names to embeddings, these will capture the associated meaning and context, which (combined with age) can be exploited as a rich feature set for training a neural network for disease incidence estimation. We compared the performance of different word embeddings in three different scenarios:

1. Where we have data on the incidence of the disease in other countries and data on the incidence of other diseases in the same country, and are simply missing data for a specific disease-country pairing.
2. Where we have data on the incidence of the disease in other countries, but no data for the country of interest.
3. Where we have data on the incidence of other diseases in that same country, but no data on the disease of interest.

## 2. Methods

### 2.1. Ethics Declarations

The analyses shown in this paper used publicly available, aggregated data and therefore ethical approval was not required.

### 2.2. Data Sources

#### 2.2.1. Global Burden of Disease Study

The Global Burden of Disease (GBD) study (20), conducted by the Institute for Health Metrics and Evaluation (IHME), aims to systematically and scientifically quantify health losses globally. The GBD dataset captures data from 195 countries globally, and combines these data to produce accurate age- and sex-specific estimates of the incidence, prevalence, and rates of disability and mortality that are caused by over 350 diseases and injuries. Data are used from many sources, including surveys, administrative data (including vital registration data, census data, epidemiological, and/or demographic surveillance data), hospital data, insurance claims data, disease registries, and other related sources. As well as data published in the scientific literature, unpublished data are sourced directly from collaborating researchers. This dataset was used primarily to develop and validate the methods used in this paper using cross-validation.

#### 2.2.2. Additional Sources Using Published Epidemiological Data

In order to investigate the ability of the deep learning models to generalize, additional data stemming from published scientific literature and national reports was used as an independent test set. These data were sourced to inform disease incidence estimates for Babylon Health's AI symptom checker, and are typically data from national statistics, disease surveillance/registries, and large-scale or population-level cohort and cross-sectional studies. It includes studies from 25 countries for 232 diseases.

### 2.3. Word Embeddings

There are many methods for learning word embeddings from text. Words are generally represented as binary, one-hot encodings which map each word in a vocabulary to a unique index in a vector. These word encodings can then be used as inputs to a machine learning model, such as a neural network, to learn the context of words. The information encoded in these embeddings is tied to the context that was used to train the neural network. Word embeddings can discover hidden semantic relationships between words and can compute complex similarity measures. If these embeddings were obtained from training on different data sources, the context encoded would likely differ. Consequently, better performance in downstream tasks will be linked to the information content encoded in these dense representations of words and its relationship with the task itself.

In this paper, we evaluated different types of word representations, obtained by different modeling strategies, on the downstream task of predicting disease incidence. This was performed by using the embeddings as inputs to a neural network for estimating disease incidence.

#### 2.3.1. Global Vectors for Word Representation

The Global Vectors for Word Representation (GloVe) model is built on the word2vec method (21), which initially converts words to numeric values. The GloVe model then learns its embeddings from a co-occurrence matrix of words, where each potential combination of words is represented as an entry in the matrix as the number of times the two words occur together within a pre-specified context window. This window moves across the entire corpus. In this work, we used the pre-trained GloVe model trained on common crawl data (17) from raw web page data. Some Publicly available information about diseases and demographics of different countries are present in such data and therefore, it is expected that such embeddings will facilitate a prediction in our model.

#### 2.3.2. BioBERT

Bidirectional Encoder Representations from Transformers (BERT) (22) is a contextualized word representation model which learns the context for a given word from the words that precede and follow it in a body of text (22). We used BioBERT, which is a model initialized with the general BERT model but pre-trained on large-scale biomedical corpora, such as PubMed abstracts and PMC full-text articles. This enables the model to learn the biomedical context of words.

#### 2.3.3. Universal Sentence Encoder

The Universal Sentence Encoder (USE) is a language model which encodes context-aware representations of English sentences as fixed-dimension embeddings.

#### 2.3.4. Feature Fusion

In addition to testing each of the language models individually, we performed feature fusion to combine the three word embeddings into a single vector by concatenation. The neural network was then trained on the combined representation.

#### 2.3.5. Sentence Embeddings

We treat the names of diseases and countries as sentences because they may consist of multiple words. The models USE and BioBERT are capable of producing embeddings for sentences and for words. GloVe and other word2vec models are limited to produce only embeddings for single words. In order to extract sentence embeddings with those models, we use the bag of words approach and computed the min, max and average values of all word embeddings within a bag of words.

#### 2.3.6. Evaluating the Contextual Information of Word Embeddings

It is important to evaluate the context that each embedding type captured, prior to using them for training disease incidence estimation models. For the embeddings to be meaningful, the word representations for either countries or diseases need to encapsulate relationships amongst each other. For instance, country embeddings for France and Spain should display similarities between each other that cover both geographical and socioeconomic metrics.

We performed two classification experiments to evaluate the context captured by the different embeddings. In these experiments, the input features were word embeddings obtained from either disease or country names whilst the classification labels were either GBD disease groups or country clusters (23). The resulting classification accuracy can serve as a metric to capture the contextual power of each embedding method when applied to either diseases or countries.

The first experiment aimed at evaluating whether disease embeddings capture context and similarities between diseases. In this experiment, we learned to predict the 17 high-level GBD disease groups (section A.6) from disease embeddings. The second experiment was focused on embeddings computed from countries and whether they can capture both geographical and economic dimensions. This can be evaluated by considering the classification of 21 country clusters, such as “High-Income Asia Pacific” and “Western Europe” from country embeddings (section A.7).

Linear Support Vector Machines (24) were trained for each experiment across a candidate set of hyperparameters. Models were trained and evaluated using 3-fold cross-validation. The cross-validation experiments were repeated ten times to mitigate any potential bias in the training and validation split. The best performing model for each embedding across both experiments were then used to assess the accuracy.

### 2.4. Training a Neural Network to Estimate Disease Incidence

**Figure 1:**
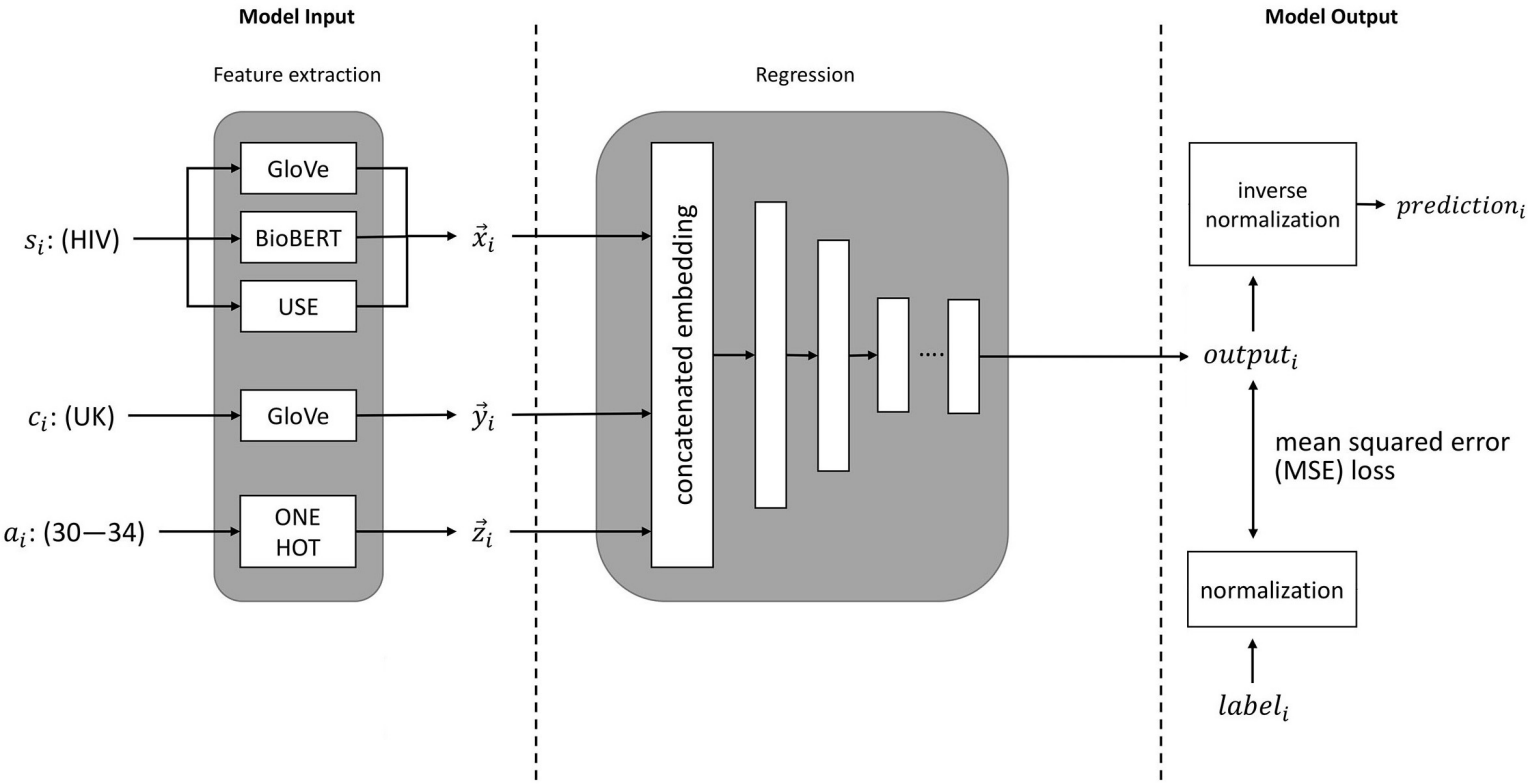
An illustration of machine learning pipeline we used for estimation of disease incidence. *s*_*i*_ represents the sentence embeddings of the disease of interest (e.g., HIV), *c*_*i*_ represents the embedding of the country of interest (e.g., UK), *a*_*i*_ represents the age group of interest (e.g., 30–34 years), and *label*_*i*_ represents the ground-truth value (from the GBD study).

The process for training a neural network to predict disease incidence rates is illustrated in Figure 1. These input features to the neural network consist of embeddings of disease, country, and age group. The neural network outputs a prediction for the incidence of a specified disease. Prior to training, the values for disease incidence are pre-processed with a log transformation. An inverse log transformation must therefore be applied to the neural network output to obtain the disease incidence rate.

#### 2.4.1. Applications of Disease Incidence Prediction

We investigated the three following incidence estimation applications:

- **Application 1—specific disease-country pairs**. This task simulates a scenario where we need to predict incidence rates for a specific diseases in a selected set of countries. This is important if data points are missing or are difficult to collect in the target country. For this application, we have data of the target disease in other countries, and data of other diseases in all countries yet data for a specific target disease-country pair is missing.
- **Application 2—previously unseen countries**. There may be cases where there is no high-quality data available in countries with poor healthcare and data infrastructure. For these situations, it may be desirable to predict incidence rates of all diseases. For this application, we simulate the case where we have no data for any disease in the target country but comprehensive incidence data for all others.
- **Application 3—previously unseen diseases**. This represents a situation where we have a key disease for which incidence data is difficult to obtain. This application consequently deals with the prediction of disease incidence rates for a given disease. In this case, incidence data is available for other diseases, but there is no data about the new, “unseen” disease in any country.

#### 2.4.2. Data Inputs

##### 2.4.2.1. Disease embeddings

We generated disease embeddings using each of the methods described in section 2.3.

##### 2.4.2.2. Country embeddings

We used the GloVe model to create representations of countries. We observed in our experiments that this model performed best for that purpose (see sections 2.3.6 and 3.2, Table 1).

**Table 1:** Classification results for GBD disease groups using disease embeddings and country clusters with country embeddings.

	**GBD disease groups**			**Country clusters**		
**Model**	**GloVe**	**BioBERT**	**USE**	**GloVe**	**BioBERT**	**USE**
Accuracy	**0.77 (0.03)**	**0.77 (0.02)**	0.66 (0.02)	**0.73 (0.02)**	0.17 (0.02)	0.62 (0.03)

*Mean (Standard deviation) of cross-validation results are reported. The numbers in bold highlight the best performing models*.

##### 2.4.2.3. Age embeddings

The 20 age groups of 5-years periods (0–4, 5–9, …, 95+) were represented as binary one-hot vectors. Representing age groups in this way means that they are treated as separate categories, so that non-linear associations between incidence and age can easily be modeled.

#### 2.4.3. Experimental Set-Up

We have used two independent and non-overlapping data sets for model development and evaluation.

The first data set is the GBD data (section 2.2.1) which was used for model development and hyper-parameter tuning. This dataset consists of 199 diseases that are annotated with incidence values across 195 countries and 20 age groups. We removed a subset of data points with zero incidence values from the original GBD study (132,903/626,580 data points, 21%). Zero incidence can happen either because data is not available or the actual incidence value is zero for some specific data entries. Since the distribution of disease incidence values was highly skewed, we log-transformed the data to base 10. The predictions from the model were inverse log-transformed to derive estimates of disease incidence.

We selected the hyperparameters of the model using 10-fold cross-validation on the GBD data. This avoids over-optimistic estimates of the model's performance, which can arise if the model is trained and tested on the same data. In each fold, we train with 90% of the GBD data, and predict on the remaining 10%. We use the following split for each application (see section 2.4):

For **Application 1**, each fold contains randomly selected country-disease pairs, where it is possible that data from the same disease or country can occur in the training and validation set but not both. This model is optimized for predicting disease incidence for country-disease combinations the model has not seen before, for example HIV in Singapore. In this example, the training data may contain disease incidence estimates for other diseases in Singapore, and for HIV in other countries. The model is therefore able to learn from these combinations of samples and then to predict the incidence for a different disease-country pair.

For **Application 2**, we ensured cross-validation was independent of the country. Within each fold of the data, the model was trained on data from 90% of countries, and validated on data from the remaining 10% of countries.

For **Application 3**, we ensured that cross-validation was independent of disease, but not country. Within each data fold, the model was trained on data from 90% of diseases, and validated on data using the remaining 10% of diseases.

We further used the published epidemiological data as the independent test set. The best performing model found and trained on the GBD data was then evaluated on this secondary dataset.

#### 2.4.4. Implementation Details

We performed a hyperparameter search on the neural networks for each of the three applications. The final neural network architecture was selected based on the overall performance across all applications using 10-fold cross validation. The resulting neural network has five hidden layers that are stacked in form of a funnel with 256, 128, 64, 16, and 4 neurons, respectively. Each layer of the neural network consisted of a fully connected layer, followed by Batch Normalization (25) and a Rectified Linear Unit (ReLU). We applied the root mean squared error as a loss function on the predicted outputs. Lastly, we used the Adam optimizer (26) with an initial learning rate of 3 × 10^−4^ and standard values for the exponential decay of moment estimates.

#### 2.4.5. Evaluation of Model Performance

##### 2.4.5.1. Baseline comparisons

For the dataset sources described in section 2.4.3 (GBD data and published epidemiological data), we investigated the performance of neural networks in predicting disease incidence across applications defined in section 2.4.1. Additionally, we compared the performance of the neural networks against three separate baselines:

1. The global average incidence (Global) for the disease of interest. This is a naïve baseline that all models should outperform.
2. A ridge regression (RidgeReg) model trained on language embeddings denoted as RidgeReg. This allows us to gauge the performance gain obtained from more complex models, such as neural networks. Note that this baseline uses BioBERT embeddings for diseases and GloVe embeddings for countries as inputs.
3. Neural network models where the input features are one-hot encoded vectors (OneHot) for countries and diseases, respectively. We considered a model with only disease labels (OneHot^*d*^), only country labels (OneHot^*c*^), and one that uses both disease and country one-hot vectors (OneHot^*d, c*^). This allows us to assess the gain obtained by using language embeddings.

##### 2.4.5.2. Metrics for evaluating performance

We used the mean absolute error (MAE) in *log*_10_ space to evaluate the performance of the disease incidence estimation. For example, a prediction with MAE of 0.2 is either 1.58 times larger or lower than the “ground truth” value. The factor of 1.58 is computed by inverse transformation (10^0.2^ = 1.58). To measure the similarity of relative rankings of the estimates (in our case, between our predictions and disease incidence values in the GBD study), we calculated the inter-group concordance ρ_*c*_ ranking whose values are bounded between 0 (worst) and 1 (best) (detailed definition can be found in section A.1 in the Appendix).

## 3. Results

We first evaluated the contextual information of the country and disease embeddings to justify their use in all following experiments (section 3.1). We then evaluated the performance of each language embedding on the three possible applications (section 3.2) and report results for both the GBD (section 2.2.1) cross-validation results and the independent test set (section 2.2.2). We also conducted additional experiments (see Appendix) including: (1) estimating the accuracy of the BioBERT feature model on the GBD data across different age groups (section A.2), (2) estimating the accuracy of the BioBERT feature model on the GBD data across various types of diseases (section A.3) and (3) demonstrating an illustrative scenario where we predicted UK disease incidence for previously unseen diseases (section A.4).

### 3.1. Contextual Information of Disease and Country Embeddings

Results for the classification experiments for GBD disease groups using disease embeddings and country clusters using country embeddings are shown in Table 1. We report the mean and standard-deviation from 10-repeated 3-fold cross validation experiments. The results indicate that GloVe and use country embeddings capture meaningful relationships between countries whilst BioBERT country embeddings are ineffective as they were trained on large-scale biomedical corpora. In contrast, all language models are able to effectively capture disease semantics with BioBERT and GloVe performing best and equitably.

### 3.2. Effect of Language Embedding on Disease Incidence Estimation

Results for the performance across various embeddings are reported for the GBD data and independent test data in Tables 2–4. Models that exploited BioBERT embeddings on average saw the best performance with consistently lower MAE and high concordance scores. However, models that employ GloVe and USE embeddings can also perform well, such as in the previously unseen countries application (Table 4) where the Fusion model performed best on average.

**Table 2:** Application 2 model performance with different input features for previously unseen diseases on the GBD and the test data.

**Data**									
**Model**	**Global**	**RidgeReg**	**OneHot^***d***^**	**OneHot^***c***^**	**OneHot^***d, c***^**	**BioBERT**	**GloVe**	**USE**	**Fusion**
**Training and Validation (from the GBD study)**									
MAE	N/A	1.03	N/A	0.805	N/A	0.781	0.807	0.765	**0.736**
ρ_*c*_	N/A	0.726	N/A	0.790	N/A	0.796	0.775	**0.826**	0.806
**Test (from the epidemiological literature)**									
MAE	N/A	1.02	N/A	N/A	N/A	**0.933**	0.989	1.01	2.43
ρ_*c*_	N/A	0.982	N/A	N/A	N/A	0.967	**0.971**	0.962	0.931

*The numbers in bold highlight the best performing models*.

**Table 3:** Application 1 model performance with different input features for specific disease-country pairs on the GBD and the test data.

**Data**									
**Model**	**Global**	**RidgeReg**	**OneHot^***d***^**	**OneHot^***c***^**	**OneHot^***d, c***^**	**BioBERT**	**GloVe**	**USE**	**Fusion**
**Training and Validation (from the GBD study)**									
MAE	0.207	0.559	0.166	0.155	**0.152**	0.157	0.157	0.168	0.157
ρ_*c*_	0.952	0.867	0.987	0.988	**0.990**	**0.990**	0.988	0.985	0.988
**Test (from the epidemiological literature)**									
MAE	N/A	1.13	N/A	N/A	N/A	**0.835**	0.910	1.06	3.78
ρ_*c*_	N/A	0.97	N/A	N/A	N/A	**0.977**	0.970	0.960	0.938

*The numbers in bold highlight the best performing models*.

**Table 4:** Application 3 model performance with different input features for previously unseen countries on the GBD and the test data.

**Data**									
**Model**	**Global**	**RidgeReg**	**OneHot^***d***^**	**OneHot^***c***^**	**OneHot^***d, c***^**	**BioBERT**	**GloVe**	**USE**	**Fusion**
**Training and Validation (from the GBD study)**									
MAE	0.212	0.562	0.204	N/A	N/A	0.197	0.198	0.209	**0.196**
ρ_*c*_	0.965	0.866	0.953	N/A	N/A	0.954	**0.955**	0.953	**0.955**
**Test (from the epidemiological literature)**									
MAE	N/A	1.12	N/A	N/A	N/A	**0.881**	0.921	1.07	0.937
ρ_*c*_	N/A	0.976	N/A	N/A	N/A	0.972	0.977	0.970	**0.978**

*The numbers in bold highlight the best performing models*.

Whilst most embedding methods produced accurate incidence estimates in the GBD dataset, it is apparent that BioBERT, followed by GloVe embeddings, produced the best results generally in the independent test set when compared to USE. For instance, BioBERT and GloVe had an MAE of 0.157 and 0.157 with concordance of 0.990 and 0.988, respectively compared to an MAE of 0.168 and a concordance of 0.985 for USE in the specific disease-country pairs application (Table 3). This illustrates that these embeddings contain informative, contextual information. This is validated in the one-hot model, which used one-hot encoded representations and suffered in performance as seen in the previously unseen diseases (Table 3) and previously unseen countries (Table 4). Note that models with one-hot features are only applicable when the target disease or country is present in the training and test data. For this reason, they do not apply to the test set because the diseases in the training and test data do not overlap.

We evaluated the utility of BioBERT embeddings by comparing the performance of the neural network method (BioBERT) with a ridge regression that used BioBERT features (RidgeReg). The neural network method saw consistently better results compared to the ridge regression across all applications for the GBD dataset.

The performance of most neural network models was consistently high in the specific disease-country pairs application (Table 3) and previously unseen countries(Table 4). However, there was a marked decrease in the validation metrics within the previously unseen diseases application (Table 3). For instance, the MAE of BioBERT rose from 0.157 (Table 2) and 0.197 (Table 4) to 0.781 whilst the concordance of GloVe for instance dropped from 0.955 (Table 3) and 0.988 (Table 4) to 0.775.

### 3.3. Performance Across Different Magnitudes of Incidence

**Figure 2:**
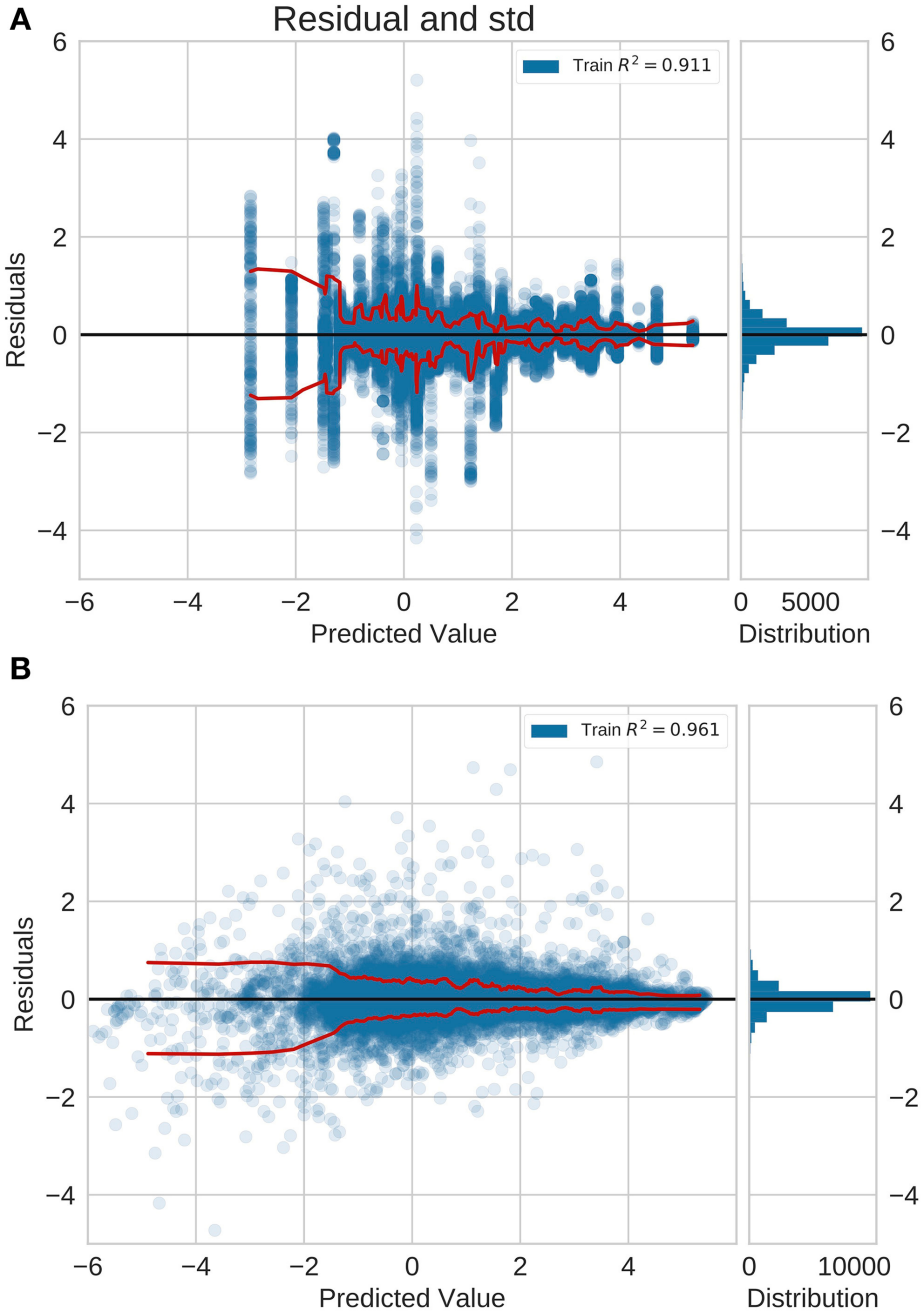
Residual plots for **(A)** naive baseline and **(B)** NN predictions with feature fusion for previously unseen countries on the GBD dataset. Red lines indicate the standard deviation. This is also the logarithm of the error. It is positive (negative) if the ML model overestimates (underestimates) the true value. The plots show that the errors for diseases with a higher incidence rate are lower.

We examined the performance of the network trained with feature fusion across diseases with different magnitudes of incidence rate. We compared the distribution of errors with the baseline model that predicted incidence rates using a global average estimate (Figure 2). Note that in these plots the Y-axis shows the MAE in log space and X-axis shows the predicted exponent of the incidence value. The incidence is normalized for a population of 100,000 people. The true incidence can be computed as follows: *I* = 10^*x*^/100, 000.

For the previously unseen countries application (Figure 2), we observed a decrease in the error magnitude at higher incidence rates across the baseline and the trained network. This illustrates that both predictive models saw higher accuracy for common diseases whilst exhibiting a reduction in performance for rare diseases. However, this effect is more pronounced in the baseline model.

**Figure 3:**
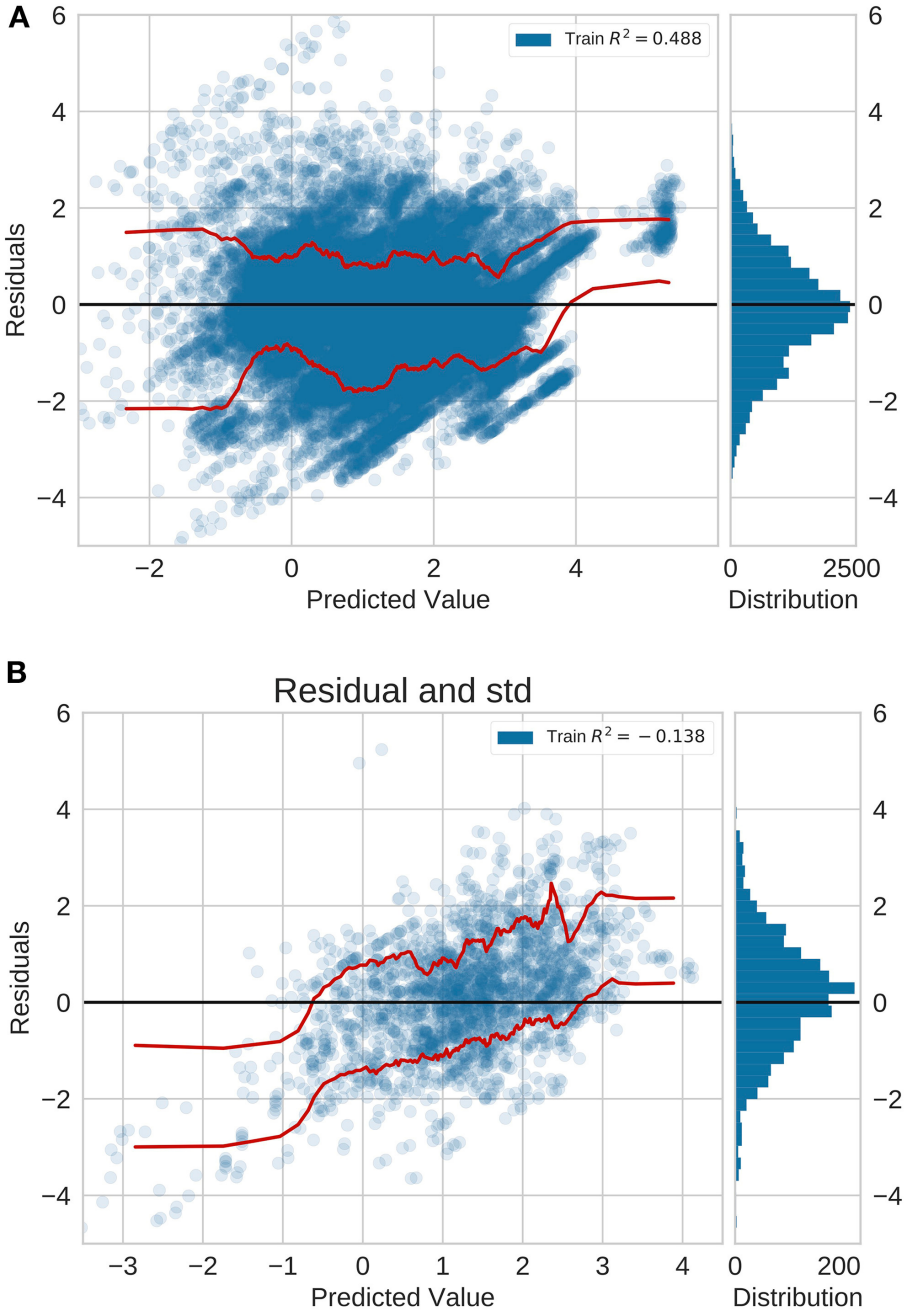
Residual plots for previously unseen diseases on data from **(A)** GBD and **(B)** peer-reviewed journals. Red lines indicate the standard deviation.

In the previously unseen diseases application using neural networks with feature fusion, we analyzed the error distribution in the GBD validation set and the independent test set with data originating from peer-reviewed literature (Figure 3). For this application, the residual distribution is much wider and the accuracy is lower for both the GBD validation set and test set. Importantly, we did not validate the global average baseline method in this application since a global average prediction can not be made if we deal with new diseases.

## 4. Discussion

In this study, we tested the ability of different language models to encode contextual information, and used the corresponding embeddings as inputs to a neural network which was used to predict disease incidence. We found that on average, models using BioBERT embeddings performed best across all metrics. We observed high performance when predicting for previously unseen countries and specific disease-country pairs, which was consistent across age groups (for more details, see section A.2). Performance for previously unseen diseases was lower, varied substantially with age, and performance was notably lower for diseases which are highly dependent on location and climate. Overall, predictions were more accurate for common diseases than rare diseases (see section A.3).

BioBERT was on average, the best-performing language model for creating disease embeddings across all three applications: predicting disease incidence for previously unseen diseases, previously unseen countries, and specific disease-country pairs. The word embeddings for BioBERT are trained with text from medical journals and other clinical literature; this model should therefore have the most relevant context for interpreting words, which should be reflected in better disease incidence estimates from the neural network using these embeddings. Interestingly for previously unseen diseases and specific disease-country pairs, using feature fusion to combine information from the three language models resulted in substantially higher MAE than using BioBERT or other language models individually when we tested our models on external data from published epidemiological literature. This suggests that using BioBERT alone results in sufficient contextual information, and further feature augmentation from other sources only adds redundant or correlated data.

When comparing our predictions for the GBD data, we observed that performance for previously unseen diseases was significantly lower than for previously unseen countries and specific disease-country pairs. The purely data-driven neural network is able to predict disease incidence better for previously unseen countries and specific disease-country pairs because it already has data for the incidence of the disease it is trying to predict, and can draw sufficient context from the country embeddings to make a prediction for a new country. However, it is difficult to fully encapsulate how a previously unseen disease is similar to other diseases within a word embedding, and so the model's predictive ability is more limited for previously unseen diseases. This reflects our general state of knowledge; we can make good inferences for disease incidence in countries where data is lacking, based on our knowledge of the country's socioeconomic situation, location, and healthcare provision, but struggle to predict the incidence of an unknown disease, regardless of how much data we have on other diseases in the same country. This is because the incidence of a disease is not only influenced by country-level factors but also by many biological, immunological, and sociodemographic factors.

We also observed discrepancies in the MAE of the predictions across the GBD study and the published epidemiological study (independent test set) yet generally equitable performance in the concordance index for disease-country pairs (Table 2) and previously unseen countries (Table 4) applications. Whilst this may suggest that the trained models have trouble generalizing, this is in fact a symptom of the inability of machine learning models to adapt to data that differs from the training data statistics. Both datasets are independent and contain certain non-overlapping sets of examples. As the test set contains new unseen examples, we face a scenario where incidence values need to be predicted for out of distribution examples. Additionally, it is a valid to assume that both datasets are drawn from different distributions as the mechanisms for generating the data differ. This is a case of distribution shift, which can negatively affect performance (27). In effect, both out of distribution examples and distribution shift are likely to negatively impact metrics, such as the MAE, which measures the average magnitude of the error. Despite this disparity, we observed equitable levels of performance when considering the inter-group concordance index (except for the application for the previously unseen diseases). This demonstrates that the ranking of the predictions is maintained despite the systematic errors introduced by the new dataset; further supporting the utility of language models and neural networks in the challenging problem of predicting disease incidence.

Deep learning methods for predicting disease incidence, which use contextual embeddings learnt from unstructured information, have the potential to give better estimates of disease incidence than are currently available for settings where high quality data is lacking. In resource poor settings, where healthcare infrastructure is weak and expressed through the lack of doctors, nurses and hospitals, it is unlikely that there is access to reliable data that facilitates estimating disease incidence (28). In these circumstances, the deployment of automated methods, such as the those presented in this paper, show the potential to benefit such populations (29).

Studies, such as the GBD, which rigorously model disease statistics using information from multiple data sources, are limited by the time lag of data becoming available, and in their ability to incorporate new conditions due to the substantial effort involved in reviewing data and building new models. Whilst the method we propose in this paper may be less rigorous, it is substantially quicker to implement for new diseases and can be easily updated to incorporate up-to-date contextual information for existing diseases. We therefore suggest it as a useful complement to existing modeling efforts, where data is required more rapidly or at larger scale than traditional methods allow for. This could be particularly useful for use in AI-driven digital health products, where the data will undergo further processing and a clinician-validated approximation of the disease incidence is adequate.

## 5. Conclusion

In this work, we developed a machine learning method based on deep learning and transfer learning. We used embeddings, which had been trained by their creators using large amounts of unstructured and freely accessible text data, to train the target neural network for incidence estimation using epidemiological data from the GBD study. We investigated three popular different language model architectures and text corpora (BioBERT, USE, GloVE) in addition to numerous baselines. We have shown that the BioBERT language model performs well at encoding contextual information relating to disease incidence. The resulting embeddings can be used as inputs to a neural network to successfully predict disease incidence for previously unseen countries and specific disease-country pairs, but is more limited in predicting for previously unseen diseases. Whilst this method is not a replacement for robust epidemiological modeling, we suggest that it could be a useful alternative when faced with situations where approximate estimates are needed rapidly on a large-scale.

## Data Availability Statement

The data analyzed in this study is subject to the following licenses/restrictions: we used data from the Global Burden of Disease study to develop and test the methods used in this paper, which is available under license from the Institute for Health Metrics and Evaluation. The additional data used for validation is available from national statistics, disease surveillance/registries, and scientific research papers. Requests to access the GBD datasets should be directed to http://www.healthdata.org/gbd.

## Author's Note

This manuscript was developed as part of research initiative at Babylon Health.

## Author Contributions

YZ: data analysis, figures, and writing. RW: data interpretation, literature search, and writing. JW: data collection, data interpretation, and writing. AB and CH: study design and writing. SL: study design and data collection. FB: data analysis and writing. SJ and YP: study design and data interpretation. YZ and RW: study design. All authors contributed to the article and approved the submitted version.

## Conflict of Interest

All authors were employed by Babylon Health.
