# Supplementary material for: Applying Artificial Intelligence Methods for the Estimation of Disease Incidence: The Utility of Language Models
Source: Front Digit Health. 2020 Dec 15;2:569261. doi: 10.3389/fdgth.2020.569261 (PMC8521977; doi:10.3389/fdgth.2020.569261)
Supplement: Supplementary file 1 [file Data_Sheet_1.pdf]

A Appendix

A.1 Definition of inter-group concordance

$$conc = \frac{\#(sign(\hat{y}_i - \hat{y}_j) == sign(y_i - y_j))}{{}^nC_2}, \forall i, j \in N, i \neq j \quad (1)$$

where ${}^nC_2$ is the number of possible combinations of choosing 2 different samples from the sets. This leads to the combinatorial explosion problem for large datasets. To avoid this, we take 1000 random sample pairs to calculate the concordance score and repeat this process 10 times to also provide the corresponding standard deviation.

A.2 Word embeddings for country classification and disease classification

A.2.1 Method

It is important to evaluate the context that each embedding type captures, prior to using them for training disease incidence estimation models. For the embeddings to be meaningful, the word representations for either countries or diseases need to encapsulate relationships amongst each other. For instance, country embeddings for France and Spain should display similarities between each other that cover both geographical and socioeconomic metrics.

To evaluate the contextual meaning of the embeddings types, we performed two classification experiments where the input features are word embeddings obtained from either disease or country names and the labels to classify are either the GBD disease groups or country clusters[22] (see Appendix). We hypothesized that the resulting classification accuracy could serve as a metric to capture the contextual power of each embedding method when applied to either diseases or countries.

The first experiment aimed at evaluating whether disease embeddings capture context and similarities between diseases. The input features are word embeddings obtained from disease names and the labels to predict are the 17 high-level GBD disease groups (Section A.6). The second experiment was focused on embeddings computed from countries and whether they can capture both geographical and economic dimensions. This can be evaluated by considering the classification of 21 country clusters such as “High-Income Asia Pacific" and “Western Europe" from country embeddings (Section A.7).

Linear Support Vector Machines[23] were trained for each classification experiment across a candidate set of model hyperparameters. Models were trained and evaluated using 3-fold cross-validation. The cross-validation experiments were repeated 10 times to mitigate any potential bias in the training and validation split. The best performing models for each embedding across both experiments were then used to assess the accuracy.

A.2.2 Results

Results for the classification experiments are reported below in Table 4. We reported the average and standard-deviation of the model accuracy across cross-validation folds. For the GBD disease group classification, we observed equitable performance across the GloVe and BioBERT disease embeddings with 0.77 accuracy whilst USE embeddings saw 0.66 accuracy.

For the country cluster classification, we observed highest performance across GloVe embeddings with 0.73 compared to 0.17 and 0.62 for BioBERT and USE respectively. This illustrates how GloVe country embeddings capture meaningful relationships between countries whilst BioBERT country embeddings are ineffective as they were trained on large-scale biomedical corpora not useful for countries.

Table 4: Classification results for GBD disease groups using disease embeddings and country clusters with country embeddings. Reported results are from 10-repeated 3-fold cross-validation experiments.

	GBD disease groups			Country clusters		
model	GloVe	BioBERT	USE	GloVe	BioBERT	USE
accuracy	**0.77 (0.03)**	**0.77 (0.02)**	0.66 (0.02)	**0.73 (0.02)**	0.17 (0.02)	0.62 (0.03)

A.3 Performance across different age groups

We evaluated the performance of the BioBERT model across all applications stratified by age-group. We quantified both the concordance and MAE between predicted incidence rates and the ground truth (Figure 4). Across the previously

unseen countries application (blue) and the specific disease-country pairs application (green), the performance was consistently high and constant across all age-groups. In contrast, in the previously unseen diseases application that aimed at predicting unknown target diseases across all countries, the MAE and the concordance varied with age-group with best performance (high concordance, low MAE) across adults and sharp drops at both extremes of the age spectrum.

A.4 Performance across different clusters of diseases

We further analysed the performance in the previously unseen diseases by evaluating the model across 17 disease groups based on the GBD model of diseases. We computed the MAE and concordance between predicted values and the ground-truth with the standard-deviation of these measures computed over the cross-validation folds (Figure 5).

The three disease groups with the highest error were: 1) neglected tropical diseases and malaria, 2)Other infectious disease and 3) nutritional deficiencies. Diseases stemming from these groups are generally difficult to predict accurately since they are highly dependent on location and climate.

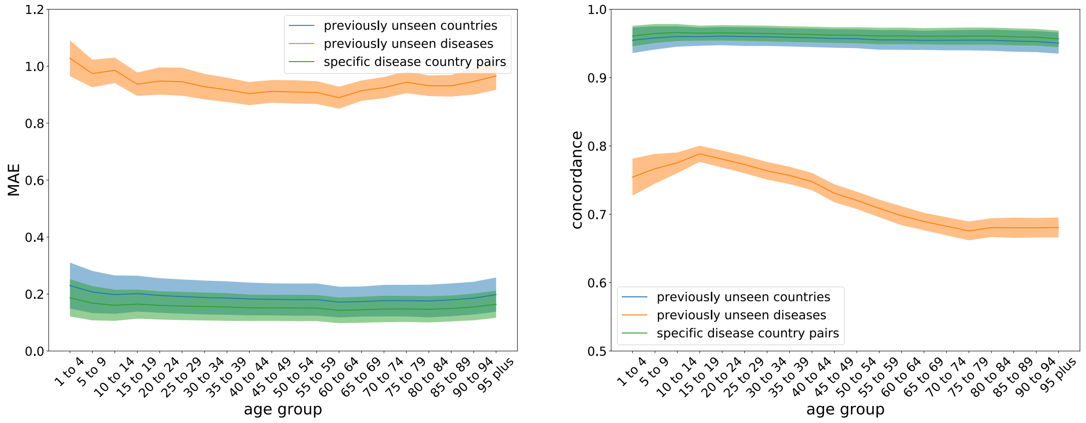

Figure 4: Performance in terms of (a) MAE and (b) concordance across different age groups. Both models for 1) specific disease-country pairs (green) and 2) previously unseen countries (blue) reach high performance because they have exposure to all disease embeddings. Predicting statistics for new diseases is much more challenging and therefore we see a lower performance on model for previously unseen diseases (orange)

A.5 GBD disease groups

- **HIV/AIDS and sexually transmitted infections**: Genital herpes, Trichomoniasis, Syphilis, Chlamydial infection, HIV/AIDS, Gonococcal infection
- **Respiratory infections and tuberculosis**:Lower respiratory infections, Tuberculosis, Upper respiratory infections, Otitis media
- **Enteric infections**: Invasive Non-typhoidal Salmonella (iNTS), Diarrheal diseases, Typhoid fever, Paratyphoid fever
- **Neglected tropical diseases and malaria**: Malaria, Leprosy, Dengue, Visceral leishmaniasis, Cutaneous and mucocutaneous leishmaniasis, African trypanosomiasis, Rabies, Zika virus, Food-borne trematodiases, Cystic echinococcosis, Chagas disease, Ebola, Guinea worm disease, Yellow fever
- **Other infectious diseases**: Encephalitis, Diphtheria, Measles, Tetanus, Varicella and herpes zoster, Acute hepatitis C, Meningitis, Acute hepatitis B, Acute hepatitis A, Whooping cough, Acute hepatitis E
- **Nutritional deficiencies**: Iodine deficiency, Protein-energy malnutrition, Vitamin A deficiency
- **Neoplasms**: Lip and oral cavity cancer, Esophageal cancer, Acute myeloid leukemia, Brain and nervous system cancer, Nasopharynx cancer, Acute lymphoid leukemia, Mesothelioma, Kidney cancer, Non-Hodgkin lymphoma, Myelodysplastic, myeloproliferative, and other hematopoietic neoplasms, Non-melanoma skin cancer (basal-cell carcinoma), Breast cancer, Testicular cancer, Bladder cancer, Chronic lymphoid leukemia, Stomach cancer, Thyroid cancer, Larynx cancer, Multiple myeloma, Liver cancer, Tracheal, bronchus, and lung cancer, Colon and rectum cancer, Other pharynx cancer, Pancreatic cancer, Chronic myeloid leukemia,

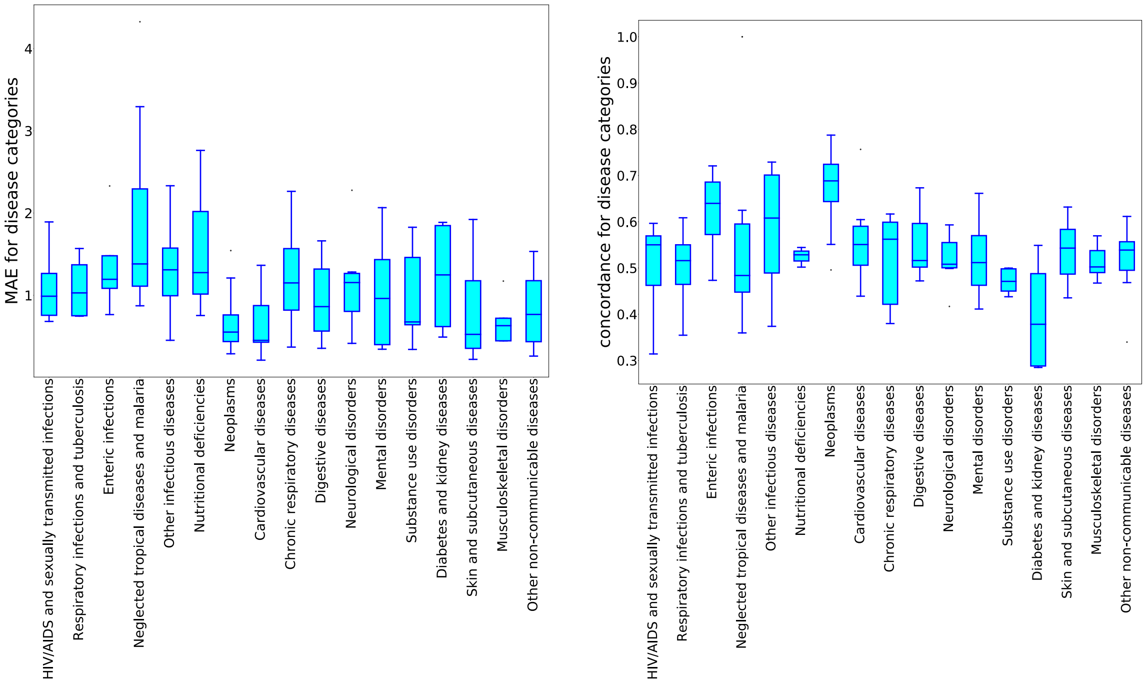

(a) MAE (b) concordance

Figure 5: Whisker plots showing the performance in terms of (a) MAE and (b) concordance across the different type of diseases. Higher errors are found on diseases that are region specific such as those of type "neglected tropical diseases and malaria".

Hodgkin lymphoma, Prostate cancer, Benign and in situ intestinal neoplasms, Non-melanoma skin cancer (squamous-cell carcinoma), Gallbladder and biliary tract cancer, Malignant skin melanoma

- **Cardiovascular diseases**: Intracerebral hemorrhage, Peripheral artery disease, Endocarditis, Subarachnoid hemorrhage, Non-rheumatic calcific aortic valve disease, Non-rheumatic degenerative mitral valve disease, Myocarditis, Atrial fibrillation and flutter, Ischemic heart disease, Ischemic stroke, Rheumatic heart disease
- **Chronic respiratory diseases**: Chronic obstructive pulmonary disease, Interstitial lung disease and pulmonary sarcoidosis, Asbestosis, Asthma, Silicosis, Coal workers pneumoconiosis
- **Digestive diseases**: Gallbladder and biliary diseases, Appendicitis, Cirrhosis and other chronic liver diseases, Peptic ulcer disease, Inguinal, femoral, and abdominal hernia, Gastritis and duodenitis, Inflammatory bowel disease, Pancreatitis, Paralytic ileus and intestinal obstruction, Vascular intestinal disorders, Gastroesophageal reflux disease
- **Neurological disorders**: Multiple sclerosis, Parkinson's disease, Epilepsy, Motor neuron disease, Alzheimer's disease and other dementias, Migraine, Tension-type headache
- **Mental disorders**: Conduct disorder, Schizophrenia, Major depressive disorder, Dysthymia, Bulimia nervosa, Bipolar disorder, Anxiety disorders, Attention-deficit/hyperactivity disorder, Anorexia nervosa
- **Substance use disorders**: Alcohol use disorders, Cannabis use disorders, Opioid use disorders, Cocaine use disorders, Amphetamine use disorders
- **Diabetes and kidney diseases**: Diabetes mellitus type 2, Acute glomerulonephritis, Diabetes mellitus type 1, Chronic kidney disease
- **Skin and subcutaneous diseases**: Acne vulgaris, Pruritus, Contact dermatitis, Atopic dermatitis, Viral skin diseases, Urticaria, Decubitus ulcer, Pyoderma, Fungal skin diseases, Alopecia areata, Cellulitis, Seborrhoeic dermatitis, Psoriasis, Scabies
- **Musculoskeletal disorders**: Gout, Rheumatoid arthritis, Low back pain, Neck pain, Osteoarthritis

- **Other non-communicable diseases**: Benign prostatic hyperplasia, Periodontal diseases, Urolithiasis, Edentulism and severe tooth loss, Urinary tract infections, Caries of deciduous teeth, Caries of permanent teeth

A.6 Country clusters

- **North Africa and Middle East**: Lebanon, Libya, Morocco, Oman, Syria, Tunisia, Palestine, Turkey, United Arab Emirates, Egypt, Algeria, Yemen, Iran, Afghanistan, Qatar, Kuwait, Bahrain, Jordan, Iraq, Saudi Arabia, Sudan
- **South Asia**: Bhutan, Pakistan, India, Nepal, Bangladesh
- **Central Asia**: Azerbaijan, Georgia, Armenia, Kazakhstan, Tajikistan, Uzbekistan, Kyrgyzstan, Mongolia, Turkmenistan
- **Central Europe**: Bosnia and Herzegovina, Czech Republic, Bulgaria, Croatia, Hungary, Montenegro, Romania, Serbia, Macedonia, Poland, Slovenia, Slovakia, Albania
- **Eastern Europe**: Belarus, Latvia, Lithuania, Moldova, Russian Federation, Ukraine, Estonia
- **Australasia**: Australia, New Zealand
- **High-income Asia Pacific**: Brunei, Japan, Singapore, South Korea
- **High-income North America**: Canada, United States, Greenland
- **Southern Latin America**: Argentina, Chile, Uruguay
- **Western Europe**: Italy, Malta, Andorra, Netherlands, Israel, United Kingdom, Norway, Portugal, Cyprus, Switzerland, Spain, Sweden, Ireland, Luxembourg, Denmark, Greece, Austria, Belgium, Finland, Germany, Iceland, France
- **Andean Latin America**: Bolivia, Peru, Ecuador
- **Caribbean**: Antigua and Barbuda, Puerto Rico, The Bahamas, Dominican Republic, Barbados, Belize, Dominica, Virgin Islands, U.S., Grenada, Guyana, Haiti, Cuba, Suriname, Saint Lucia, Saint Vincent and the Grenadines, Trinidad and Tobago, Jamaica, Bermuda
- **Central Latin America**: Colombia, Costa Rica, El Salvador, Honduras, Mexico, Guatemala, Nicaragua, Panama, Venezuela
- **Tropical Latin America**: Brazil, Paraguay
- **East Asia**: China, North Korea, Taiwan
- **Oceania**: Kiribati, Marshall Islands, Fiji, Northern Mariana Islands, Federated States of Micronesia, Papua New Guinea, Solomon Islands, Samoa, Tonga, Vanuatu, American Samoa, Guam
- **Southeast Asia**: Cambodia, Laos, Philippines, Maldives, Indonesia, Myanmar, Vietnam, Malaysia, Sri Lanka, Timor-Leste, Thailand, Seychelles, Mauritius
- **Central Sub-Saharan Africa**: Angola, Central African Republic, Congo, Democratic Republic of the Congo, Equatorial Guinea, Gabon
- **Eastern Sub-Saharan Africa**: Somalia, Djibouti, Uganda, Tanzania, Burundi, Comoros, Madagascar, Ethiopia, Eritrea, Rwanda, South Sudan, Zambia, Kenya, Mozambique, Malawi
- **Southern Sub-Saharan Africa**: Botswana, South Africa, Swaziland, Lesotho, Zimbabwe, Namibia
- **Western Sub-Saharan Africa**: Guinea-Bissau, Liberia, Mauritania, Mali, Niger, Sierra Leone, Togo, Guinea, Senegal, Sao Tome and Principe, Nigeria, Benin, Burkina Faso, Cameroon, Chad, Cape Verde, "Cote dIvoire", The Gambia, Ghana
